# The effect of pneumovesicoscopic Cohen surgery using a segmented submucosal tunnel and reduced intra-abdominal pressure for the treatment of vesicoureteral reflux in children

**DOI:** 10.3389/fped.2025.1709328

**Published:** 2025-12-12

**Authors:** Hong Chen, Xu Cui, Chaoming Zhou, Jianqin Zhang, Liu Chen

**Affiliations:** Department of Urology, Fujian Children’s Hospital (Fujian Branch of Shanghai Children’s Medical Center), College of Clinical Medicine for Obstetrics & Gynecology and Pediatrics, Fujian Medical University, Fuzhou, Fujian, China

**Keywords:** children’s bladder surgery, vesicoureteral reflux, pneumovesicoscopic Cohen surgery, infants, retrospective studies

## Abstract

**Objective:**

This study aims to explore the effect of using a segmented submucosal tunnel and reducing intra-abdominal pressure in the treatment of CO_2_ leaking from the bladder into the abdominal cavity in vesicoureteral reflux (VUR) patients during pneumovesicoscopic Cohen surgery.

**Method:**

A retrospective analysis was conducted on children aged 0–1 years with VUR who underwent pneumovesicoscopic Cohen surgery at the Department of Urology, Fujian Children's Hospital from 1 May 2021 to 1 October 2024. Among them, 56 cases had CO_2_ leakage from the bladder into the abdominal cavity during the surgery. The patients were divided into two groups based on the surgical method: Group A, 28 cases, where a submucosal tunnel was directly established during surgery, and Group B, 28 cases, where a submucosal tunnel was established in segments and decompression of the abdominal cavity was performed during surgery. Intraoperative and postoperative outcomes, complications, and clinical efficacy were compared between the two groups.

**Results:**

No complications from bladder leakage, anastomotic stenosis, or urinary retention occurred in either group after surgery, and no reoperations were necessary. In Group A, two cases were converted to open surgery, while no conversions were necessary in Group B. No statistically significant difference was found in indicators such as placement of the ureteral catheter, pain score (6 h after surgery), postoperative hospitalization time, conversion to open reimplantation, postoperative UTI, postoperative VUR, and postoperative hematuria time. The operation time (187.11 ± 55.29 min vs. 157.79 ± 44.96 min, *P* < 0.05) and pain score 2 h after surgery (2.21 ± 0.79 vs. 1.39 ± 0.99, *P* < 0.05) in Group B were lower than those in Group A.

**Conclusion:**

Reducing intra-abdominal pressure combined with segmental submucosal tunneling is a simple, safe, and effective approach, which is recommended for managing CO_2_ leakage from the bladder into the abdominal cavity of VUR patients during pneumovesicoscopic Cohen surgery.

## 1 Introduction

Vesicoureteral reflux (VUR) is a common congenital disease of the urinary system in children with an incidence rate of approximately 1%–2%, and one of the most common causes of febrile urinary tract infection (fUTI) (1, 2). VUR is the main cause of reflux nephropathy. Without active intervention, 10%–20% of children with reflux nephropathy will eventually develop hypertension and end-stage renal disease. The main goal of VUR treatment is to eliminate reflux, reduce the recurrence of urinary tract infection (UTI), and protect renal function. Surgical treatment is one of the main treatment methods (3–6) and mainly involves adjusting the ratio of tunnel length to ureteral diameter to 4:1–5:1 (7). Pneumovesicoscopic Cohen surgery is one of the two main surgical techniques used in China (8, 9) and is performed using an intravesical approach. The optional bladder approach surgery is challenging in children due to their small bladder volume, which results in limited surgical space and increased operational difficulty. This is particularly evident in infants under 1 year old, whose bladder volume is even smaller. If an air-filled bladder is established during surgery and CO_2_ from the bladder leaks into the abdominal cavity, bladder dilation can be affected, further narrowing the operating space and increasing surgical complexity. We found that a segmented approach to a submucosal tunnel and reduced intra-abdominal pressure can make the surgery smoother. This study retrospectively analyzes cases of VUR patients treated with pneumovesicoscopic Cohen surgery, focusing on the impact of a segmented approach to a submucosal tunnel and reduced intra-abdominal pressure on the surgery.

## 2 Materials and methods

### 2.1 Patients

A retrospective analysis was conducted on children aged 0–1 years with VUR who underwent pneumovesicoscopic Cohen surgery at the Department of Urology, Fujian Children's Hospital from 1 May 2021 to 1 October 2024. Among them, 56 cases had CO_2_ leakage from the bladder into the abdominal cavity during the surgery. The patients were divided into two groups based on the surgical method: Group A, 28 cases, where a submucosal tunnel was directly established during surgery, and Group B, 28 cases, where a submucosal tunnel was established in segments and decompression of the abdominal cavity was performed during surgery. There was no significant difference between the two groups in age, weight, sex, surgical side, or the preoperative voiding cystourethrogram (VCUG) grade (*P* > 0.05), indicating that both groups were comparable. Most patients in Group A underwent surgery between 2021 and 2023, whereas those in Group B underwent surgery between 2023 and 2024. The follow-up time of Group B was shorter than that of Group A, and this difference was statistically significant (*P* < 0.05) (Table 1). This study was conducted in accordance with the Declaration of Helsinki and with approval from the Ethics Committee of Fujian Children's Hospital (Ethics No. 2025ETKLRK06002).

**Table 1:** Patients’ characteristics and preoperative data of VUR.

Characteristics	Group A (*n* = 28)	Group B (*n* = 28)	*t*/*χ*^2^/*U*	*P*
Age at surgery (months)	9.75 ± 2.50	10.54 ± 2.30	473	0.148
Weight at surgery	9.43 ± 1.52	10.13 ± 1.38	−1.795	0.078
Male/female	19/9	23/5	1.524	0.217
Surgical side				
Left	4	4	–	0.923
Right	5	7		
Bilateral	19	17		
Preoperative VCUG grade				
Ⅰ	1	1	–	0.590
Ⅱ	4	6		
Ⅲ	12	8		
Ⅳ	22	26		
Ⅴ	8	4		
Following time (months)	29.82 ± 12.66	13.68 ± 7.37	124	0.000

Inclusion criteria: (1) children diagnosed with primary VUR in children; (2) those who complied with VUR surgical indications (10); (3) those who agreed to a pneumovesicoscopic Cohen surgery, but with an abdominal leakage during the operation. Exclusion criteria: (1) children with secondary or concurrent urinary system abnormalities, such as neurogenic bladder, posterior urethral valve, and duplicated kidney; (2) those who did not undergo surgical treatment or who cannot tolerate surgery; (3) and those undergoing other surgical procedures.

### 2.2 Surgical techniques

An experienced pediatric urologist (with decades of practice and an annual caseload of dozens of Cohen procedures since 2015) served as the primary surgeon for all cases in this study.

#### 2.2.1 Group A surgical techniques

Under direct visualization with a cystoscope, three trocars were inserted approximately 3 cm below the navel and securely fixed. The mucosa was cauterized 0.3 cm around the ureteral orifice with an electric coagulation hook. The ureter was electrically dissected intravesically under traction until it could be placed tension-free on the opposite side. The planting distance was measured according to the principle of “5:1.” Approximately 1.5 cm above the contralateral ureteral orifice was selected as the implantation site. A sufficiently wide tunnel was made between the mucosa and the muscle layer with a 3 mm shear. The tunnel was subtly dissected toward the original ureterostoma area. The ureter was then pulled through the prepared submucosal tunnel and fixed to the bladder muscle at the wall passage. Finally, the neo-ostium was sutured to the surrounding mucosa. For bilateral replantation, the lateral side with a higher VUR grade was placed upwards. The lower ureter traverses the submucosa of the trigone to the contralateral ureterostoma area. The ureteral opening and bladder mucosa were sutured intermittently with 5-0 absorbable sutures with four to six stitches. We did not routinely use indwelling double J tubes or ureteral catheters after surgery (11).

#### 2.2.2 Group B surgical techniques

The surgical procedures for Group B are basically the same as those for Group A.

##### 2.2.2.1 A segmented approach to a submucosal tunnel

When establishing a submucosal tunnel, it is estimated that the bladder mucosa will be cut open at the middle section of the submucosal tunnel between the original ureteral orifice and the new ureteral orifice, with a length of approximately 1.0 cm. The submucosal tunnel is constructed in segments: from the new ureteral orifice to the middle section of the tunnel and from the middle section of the tunnel to the original ureteral orifice. The ureter is then pulled in segments to the new ureteral orifice.

##### 2.2.2.2 Reduced intra-abdominal pressure

An incision is made at the navel, and a gastric tube (type: 10–14) is inserted into the abdominal cavity, with the opening at the other end, to relieve intra-abdominal pressure.

### 2.3 Follow-up

Postoperative reexamination of urinalysis and color Doppler ultrasound should be performed at 1, 3, and 6 months after surgery, followed by annual color Doppler ultrasound thereafter. Any postoperative fever requires urinalysis to rule out UTI. If a febrile UTI occurs postoperatively, the VCUG should be performed during follow-up examination 6 months after surgery.

### 2.4 Statistical methods

The statistical analysis was performed using SPSS 23.0 software. Quantitative data are presented as mean ± standard deviation. The independent sample *t*-test was used for normally distributed measurement data, while the Mann–Whitney *U* test was used for non-normally distributed measurement data. The chi-square test or Fisher's exact test was used for between-group comparison of the qualitative data, according to the sample size. A *P*-value of <0.05 was considered statistically significant.

## 3 Results

There were no complications such as bladder leakage, anastomotic stenosis, or urinary retention in either group after surgery, and no reoperations were required. Pain score after surgery was evaluated using the FLACC scale (12). No statistically significant difference was found in indicators such as placement of the ureteral catheter, pain score at 6 h after surgery, postoperative hospitalization time, conversion to open reimplantation, postoperative UTI, postoperative VUR, and postoperative hematuria time. In Group A, two cases were converted to open surgery, while no conversions were required in Group B. Postoperative infections occurred in six cases, with two cases checking VCUG after surgery, showing one single-sided Grade 1 VUR and the other single-sided Grade 2 VUR. The operation time (187.11 ± 55.29 min vs. 157.79 ± 44.96 min, *P* < 0.05) and pain score 2 h after surgery (2.21 ± 0.79 vs. 1.39 ± 0.99, *P* < 0.05) in Group B were all significantly lower than those in Group A (Table 2).

**Table 2:** Patients’ intraoperative, postoperative, and follow-up data.

Item	Group A	Group B	*t*/*χ*^2^/*U*	*P*
Operation time (min)	187.11 ± 55.29	157.79 ± 44.96	258	0.028
Placement of ureteral catheter	2	5	–	0.422
Pain score 2 h after surgery	2.21 ± 0.79	1.39 ± 0.99	212	0.001
Pain score 6 h after surgery	0.64 ± 1.06	0.5 ± 0.84	374	0.715
Postoperative hospitalization (d)	8.00 ± 2.46	7.61 ± 2.25	350.5	0.483
Postoperative hematuria (d)	2.39 ± 0.88	1.96 ± 0.64	286.5	0.055
Converted to open reimplantation	2	0	–	0.491
Postoperative UTI	3	3	–	1.000
Postoperative VUR	0	2	–	0.491

## 4 Discussion

VUR is a common cause of UTI in children, and surgical treatment is one of the main treatment methods, through both intravesical and extravesical approaches (10). Cohen's procedure is the most commonly used technique for an intravesical approach, representing the gold standard for VUR surgical treatment. In 2005, Yeung et al. (13) first reported the use of pneumovesicoscopic Cohen's surgery (using CO_2_) for the treatment of VUR, with a success rate of 93%–96%, comparable to open surgery (14). At present, pneumovesicoscopic Cohen's surgery remains the preferred surgery for pediatric urologists to treat VUR (2). This surgical method offers several advantages, such as minimal damage to the bladder muscle layer, reduced risk of ureteral twisting or excessive bending, a long submucosal tunnel, good anti-reflux outcomes, and few complications. However, it has certain disadvantages, including limited operating space, changing of the normal course of the ureter, and difficulty in performing subsequent retrograde ureteral procedures (15–17).

Compared with adult VUR patients, children—especially those under 1 year old—have a relatively small bladder capacity. The bladder capacity prediction formula under 1 year old is weight (kg) × 7 mL (18). A smaller bladder leads to a narrow surgical operating space, limiting the operable angle of the forceps. To effectively establish the anti-reflux mechanism, the submucosal tunnel should be 4–5 times the diameter of the ureter (7). Gas leakage into the perivesical space during the operation further reduces the bladder volume, increases surgical difficulty, prolongs the operation time, or may necessitate conversion to open surgery. Leakage is not uncommon, and the possible reasons for air leakage include the following:

(1) An expanded bladder creating a recess of the intraperitoneal area between the bladder and the abdominal wall: when the bladder is inflated and a trocar is inserted, the fixed line or trocar passing through the abdominal wall passes through this depression.

(2) Extravesical spread of gas through the ureteral hiatus after freeing the ureter (19)

(3) Trocar dislodgement during reinsertion.

(4) Thin bladder walls of infants and young children and using excessive bladder pressure which can easily lead to air leakage.

During the surgery, intraoperative gas leakage reduced the bladder space, leading to a narrow intravesical operating space and increased operative difficulty. For the two patients whose surgery was difficult to perform due to gas leakage from the bladder into the abdominal cavity, conversion to open surgery was performed in the early stages. Then we used reduced intra-abdominal pressure, allowing the bladder to expand and ensuring sufficient surgical space, facilitating a smooth surgery. Although abdominal decompression reduces the impact of gas leakage, the bladder still does not achieve the same level of expansion as it would without gas leakage. Due to issues with the operating angle and space, it was difficult to establish a long submucosal tunnel, which often leads to damage to the bladder mucosa and bladder muscle layer. And the insufficient bladder distension also resulted in inadequate bladder wall tension, increasing the difficulty of dissecting the submucosal tunnel and the damage to the bladder muscle layer. By adopting a segmented approach for establishing the submucosal tunnel, we can reduce the length of a single tunnel, decrease the angle of the operating forceps during tunnel construction, ensure visibility during operation, make the operation more convenient, and reduce damage to the bladder muscle layer and mucosa. Although an additional navel incision is made in this technique, the incision is hidden and esthetically pleasing. When using the segmented method, we did not have any surgeries that required conversion to open surgery.

Although segmental submucosal tunnel creation adds one mucosal incision and reducing intra-abdominal pressure also adds one navel incision, suturing the incisions during surgery does not significantly increase the operation time. Instead, it can overall shorten the surgical time without affecting the outcome or increasing complications or impacting prognosis. The results of this study indicate that reducing intra-abdominal pressure and segmental submucosal tunnel creation can reduce postoperative pain and shorten the operation time, thereby alleviating the suffering of children. Postoperative pain reduction is primarily attributed to the following factors: (1) In Group A, the gas that leaked into the abdominal cavity during the procedure was not expelled postoperatively, leading to gastrointestinal disturbances and subsequent pain. In contrast, Group B underwent abdominal decompression to remove the intra-abdominal gas. (2) In Group A, insufficient bladder distension resulted in inadequate bladder wall tension, which increased the difficulty of dissecting the submucosal tunnel. Consequently, Group A sustained more significant damage to the bladder muscle layer than Group B. Postoperative infections occurred in six cases, with two cases requiring VCUG follow-up after surgery, showing mild VUR. Regular follow-ups were conducted, and no recurrence of fever was observed. According to the long-term follow-up results by Gerwinn et al. (20), single FUTI after surgery mainly occurs in the first year and does not recur. In this study, four cases experienced a single FUTI within 3 months after surgery, with negative VCUG rechecks and no recurrence thereafter.

The weakness of this study is the small number of cases, single center, and the absence of details such as the threshold for performing decompression, the actual intra-abdominal pressure, and how gas-leak severity, which require more research to confirm the findings. We did not adjust for multiple comparisons in the analysis of the nine outcomes. While this increases the possibility of Type I errors (false positives), we believed it was a necessary tradeoff to reduce the risk of Type II errors (false negatives) and to avoid overlooking potentially clinically important findings in this study. Therefore, the results require validation in future prospective studies.

## 5 Conclusions

Reducing intra-abdominal pressure and segmental submucosal tunnel creation is a simple, safe, and effective method. It can be applied to children in that CO_2_ from the bladder leaks into the abdominal cavity to bladder expansion to provide sufficient surgical space and ensure the smooth progress of the surgery. This makes the surgery more convenient, reduces the difficulty of the operation, and lessens the pain of the children. At the same time, it does not increase complications and does not affect the success rate of surgery.

## Data Availability

The raw data supporting the conclusions of this article will be made available by the authors, without undue reservation.
